# Clinical outcomes associated with guideline-discordant management of asymptomatic bacteriuria and urinary tract infection in hospitalized patients with neurogenic bladder

**DOI:** 10.1017/ash.2022.348

**Published:** 2022-12-09

**Authors:** Margaret A. Fitzpatrick, Marissa Wirth, Jimmy Nguyen, Katie J. Suda, Frances M. Weaver, Stephen Burns, Eileen Collins, Nasia Safdar, Ursula Patel, Charlesnika T. Evans

**Affiliations:** 1 Rocky Mountain Regional VA Medical Center, Aurora, Colorado; 2 University of Colorado Anschutz Medical Campus, Aurora, Colorado; 3 Center of Innovation for Complex Chronic Healthcare, Edward Hines, Jr. VA Hospital, Hines, Illinois; 4 Center for Health Equity Research and Promotion, VA Pittsburgh Healthcare System, Pittsburgh, Pennsylvania; 5 University of Pittsburgh School of Medicine, Pittsburgh, Pennsylvania; 6 Loyola University Chicago Parkinson School of Health Sciences and Public Health, Maywood, Illinois; 7 VA Puget Sound Healthcare System, Seattle, Washington; 8 University of Washington School of Medicine, Seattle, Washington; 9 University of Illinois at Chicago College of Nursing, Chicago, Illinois; 10 University of Wisconsin–Madison School of Medicine and Public Health, Madison, Wisconsin; 11 William S. Middleton VA Hospital, Madison, Wisconsin; 12 Northwestern University Feinberg School of Medicine, Center for Health Services and Outcomes Research, Chicago, Illinois

## Abstract

**Objective:**

To compare clinical outcomes associated with appropriate and inappropriate management of asymptomatic bacteriuria (ASB) and urinary tract infection (UTI) among inpatients with neurogenic bladder (NB).

**Design:**

Multicenter, retrospective cohort.

**Setting:**

The study was conducted across 4 Veterans’ Affairs hospitals.

**Participants:**

The study included veterans with NB due to spinal cord injury or disorder (SCI/D), multiple sclerosis (MS), or Parkinson’s disease (PD) hospitalized between January 1, 2017, and December 31, 2018, with diagnosis of ASB or UTI.

**Interventions:**

In a medical record review, we classified ASB and UTI diagnoses and treatments as appropriate or inappropriate based on national guidelines.

**Main outcome measures:**

Frequencies of *Clostridioides difficile* infection, acute kidney injury, 90-day hospital readmission, postculture length-of-stay (LOS), and multidrug-resistant organisms in subsequent urine cultures were compared between those who received appropriate and inappropriate management.

**Results:**

We included 170 encounters with ASB (30%) or UTI (70%) diagnoses occurring for 166 patients. Overall, 86.1% patients were male, 47.6% had SCI/D and 77.6% used bladder catheters. All ASB encounters had appropriate diagnoses, and 96.1% had appropriate treatment. In contrast, 37 UTI encounters (31.1%) had inappropriate diagnoses and 61 (51.3%) had inappropriate treatment, including 30 encounters with true ASB. Among patients with SCI/D or MS, appropriate ASB or UTI diagnosis was associated with a longer postculture LOS (median, 14 vs 7.5 days; *P* = .02). We did not detect any significant associations between appropriate versus inappropriate diagnosis and treatment and other outcomes.

**Conclusions:**

Almost one-third of UTI diagnoses and half of treatments in hospitalized patients with NB are inappropriate. Opportunities exist to improve ASB and UTI management in patients with NB to minimize inappropriate antibiotic use.

Chronic neurologic disorders, such as spinal cord injury or disorder (SCI/D), multiple sclerosis (MS), and Parkinson’s disease (PD), are associated with long-term bladder dysfunction involving loss of bladder sensation and impaired storage and emptying, a condition termed neurogenic bladder (NB).^1^ Many patients with NB require the use of urethral or suprapubic catheters to assist with bladder management. The most common complications related both to NB and to catheter use are chronic bacteriuria and urinary tract infection (UTI). In the United States, UTI is the fifth most common healthcare-associated infection, accounting for ∼10% of infections in acute-care hospitalized patients,^2^ and it is the most common infection in patients with NB.^3,4^

In patients with NB, urinary stasis and catheter use along with physiologic changes in the bladder lead to chronic bladder colonization with bacteria without clear tissue invasion or true infection, termed asymptomatic bacteriuria (ASB).^5^ Establishing a diagnosis of UTI requires the presence of both clinical signs and symptoms and significant bacteriuria.^5,6^ Because of the high ASB prevalence in patients with NB^7,8^ and atypical symptoms resulting from impaired bladder sensation, UTI diagnosis and management can be challenging in this patient population.

No clinical guidelines address UTI management in patients with NB; however, best practices have been adapted from Infectious Diseases Society of America (IDSA) guidelines targeting catheter-associated (CA-)UTI^6^ and ASB^5^, as well as recommendations from the National Institute on Disability and Rehabilitation Research (NIDRR) for patients with SCI/D.^9^ In a patient with clinically significant bacteriuria, all guidelines emphasize that UTI (vs ASB) is diagnosed based on the presence of symptoms and signs of UTI. The guidelines also emphasize that (1) antibiotic choice for UTI treatment should be guided by urine culture and susceptibilities; (2) antibiotics should penetrate the urinary tract with efficacy for UTI; (3) duration of antibiotics should range from 7 to 14 days, and (4) ASB should not routinely be treated. Despite these guidelines, misdiagnosis of ASB as UTI and inappropriate treatment of appropriately diagnosed UTI are leading causes of antibiotic overuse, particularly in hospitalized patients, with up to 82% of patients with ASB receiving inappropriate antibiotics.^10–13^ Antibiotic use has been linked to adverse outcomes such as bacterial resistance, drug reactions, *Clostridioides difficile* infection (CDI), and prolonged hospital length of stay (LOS)^13–15^; however, previous studies have not specifically examined outcomes in patients with NB and suspected UTI. Furthermore, patients with NB have high risk for recurrent ASB and UTI and high rates of hospitalization, so there may be frequent opportunities to improve inpatient ASB and UTI management in this population. In a national cohort study, one-third of patients with NB due to SCI/D or MS were hospitalized over a 1-year period.^4^ Therefore, we sought to describe ASB and UTI diagnosis and treatment in hospitalized veterans with NB and compare clinical outcomes associated with appropriate versus inappropriate ASB and UTI diagnosis and treatment.

## Methods

### Study design, setting, and population

This multicenter, retrospective cohort study included veterans with NB admitted to 4 Veterans’ Affairs (VA) facilities between January 1, 2017, and December 31, 2018. All adult patients (aged ≥18 years) with NB due to underlying SCI/D, MS, or PD with an ASB or UTI diagnosis occurring during an inpatient or long-term care facility (LTCF) stay at Edward Hines, Jr, VA Hospital in Hines, Illinois, James A. Haley Veterans’ Hospital in Tampa, Florida, VA Puget Sound Healthcare System in Seattle, Washington, or the William S. Middleton Memorial Veterans Hospital in Madison, Wisconsin, were eligible for inclusion. Of these 4 sites, 3 have VA SCI/D centers that serve as referral centers for large geographic areas and that provide acute SCI/D rehabilitation and life-long follow-up care. Veterans’ Health Administration guidelines outline care for persons with SCI/D and recommend a urinalysis and urine culture as part of an annual physical checkup at an SCI/D Center for all veterans with SCI/D, regardless of the presence of UTI signs or symptoms.^16^ Also, 3 of these sites are affiliated with the national VA PD care network (ie, Parkinson’s Disease Research, Education, and Clinical or Consortium Centers), and all 4 sites are affiliated with the national VA MS care network (ie, MS Centers of Excellence). The institutional review board at the Edward Hines, Jr, VA Hospital approved this study.

### Data sources and study cohorts

Clinical and encounter data were obtained from the VA Corporate Data Warehouse (CDW), which includes clinical and administrative electronic medical record data from the Veterans’ Health Administration. Data were also obtained from the VA SCI/D registry, a national data registry for veterans with SCI/D, MS with spinal cord involvement, and amyotrophic lateral sclerosis (ALS). Patients with ALS were not included in this study. Combinations of *International Classification of Diseases, Tenth Edition, Clinical Modification* (ICD-10-CM) codes and data from the CDW and SCI/D Registry were used to identify patients with SCI/D, MS, and PD, as summarized in Supplementary Table 1.17 Among these patients with SCI/D, MS, or PD, we identified patients with NB primarily by at least 2 ICD-10-CM codes for NB or overactive bladder at least 2 months apart.^4^ A secondary NB definition was used for patients not meeting the ICD-10-CM criteria, which was the presence of at least 2 prescriptions for urinary catheter supplies during the study period.

Among the cohort of patients with NB, ASB and UTI encounters were primarily identified using ICD-10-CM codes. Because most providers may not routinely enter diagnostic codes for ASB, an additional method was also used to identify patients with ASB encounters. We included any patient with a urine culture with ≥10^5^ colony-forming units (CFU)/mL of ≥1 bacterial species who did not have a UTI ICD-10-CM code associated with an encounter ± 7 days from urine culture collection. In total, 1,246 unique patients with NB due to SCI/D, MS, or PD had 5,961 unique encounters with an ASB or UTI diagnosis during the study period. As part of a larger analysis of ASB and UTI management in multiple care settings, a stratified sample of 300 ASB and UTI encounters was identified for medical record review. This sample contained proportions of patients with SCI/D, MS, and PD that mimicked the frequencies observed in the larger cohort, but it was randomly selected otherwise. Patient-reported signs and symptoms, bladder management, and provider type and specialty (defined as the primary provider making the ASB and UTI diagnosis and treatment decisions) were recorded. Our medical record review tool was informed by the National Institute of Neurological Disorders and Stroke (NINDS) “Common Data Elements (CDEs)” for SCI,^18^ which standardize data collection and reporting among investigators. Initial reviews were conducted by a research assistant trained in ASB and UTI management and the IDSA and NIDRR guidelines. The principal investigator (PI) pilot-tested the tool in conjunction with the assistant and reviewed the first 20 records to verify accuracy. Once the assistant completed the first round of reviews, a second review was performed by the PI to assign ASB and UTI diagnosis and treatment categorizations. All data from the chart review were entered in a secure REDCap database. In total, 166 patients from the medical record review sample had 170 hospital or long-term care facility (LTCF) encounters with an ASB or UTI diagnosis, and these patients constitute the final study cohort reported here.

### Study definitions

Each of the 170 patient encounters was evaluated for appropriateness of diagnosis and treatment based on national clinical guidelines from IDSA and NIDRR.^6,9^ Diagnosis categorizations are summarized in Supplementary Table 2. Treatments prescribed for ASB and UTI diagnoses were also recorded from medical record review and were evaluated for appropriateness according to clinical guidelines. Treatment categorizations are summarized in Supplementary Table 3. Patients with inappropriate UTI diagnosis (true ASB) were analyzed in the ASB treatment group.

### Clinical outcomes

Clinical outcomes evaluated in this study were measured from the index diagnosis date and included CDI within 90 days, acute kidney injury (AKI) within 21 days, isolation of a multidrug-resistant organism (MDRO) on subsequent urine culture within 90 days, and hospital readmission within 90 days. Additionally, we recorded post–urine-culture length-of-stay (LOS). CDI was defined as a positive stool test for toxin-producing *C. difficile* and receipt of oral vancomycin, fidaxomicin, or metronidazole within 72–96 hours of the positive test result. AKI was defined as an increase in serum creatinine of ≥0.3 mg/dL for 2 consecutive measurements obtained over a 48-hour period or an increase in serum creatinine to ≥1.5 times baseline within the prior 7 days.^19^ Any MDRO on a subsequent urine culture in 90 days was recorded as an outcome, regardless of a patient’s prior positive culture for that or any other MDRO. MDROs were defined using the European Centre for Disease Prevention and Control and the Centers for Disease Control and Prevention consensus definitions proposed by Magiorakos et al^20^ and included methicillin-resistant *Staphylococcus aureus* (MRSA), vancomycin-resistant *Enterococcus* (VRE), extended-spectrum β-lactamase–producing (ESBL) Enterobacterales, multidrug-resistant *Pseudomonas aeruginosa*, and multidrug-resistant *Acinetobacter baumannii* complex.^20^ Carbapenem-resistant Enterobacterales (CRE) were defined based on the VA CRE definition.^21^

### Statistical analysis

Descriptive statistics summarized patient demographic and clinical characteristics, diagnosis and treatment categorizations, and clinical outcomes. Unadjusted logistic regression and the Mann-Whitney *U* test were used to test associations between inappropriate ASB and UTI diagnosis and treatment and outcomes in the subgroups of patients with SCI/D or MS because these groups represented the most frequent underlying neurologic diagnoses causing NB in our study cohort.

## Results

Patient and encounter characteristics are listed in Table 1, both overall and by underlying neurologic diagnosis. Overall, 86.1% of patients were male, 47.6% had SCI/D, and 77.6% used bladder catheters, of which 44.1% were indwelling urethral catheters. Also, 115 patients (91.2%) were seen in inpatient care settings, and 89 (52.4%) were seen by physical medicine and rehabilitation (PM&R) or SCI/D physicians. The cohort had a high frequencies of comorbid diabetes (30.6%), chronic kidney disease (27.1%), and chronic obstructive pulmonary disease (COPD, 20.6%). Patient characteristics were similar between SCI/D and MS groups; however, compared to patients with SCI/D or MS, patients with PD were older, had more frequent comorbidities, and had lower frequency of catheter use for bladder management. Encounters for patients with SCI/D had greater frequency of advanced practice providers and PM&R and SCI specialists compared to MS and PD, with most PD patients being seen by Internal Medicine providers.

**Table 1.** Patient and Encounter Characteristics for Patients With Neurogenic Bladder (NB) Due to Spinal Cord Injury or Disorder (SCI/D), Multiple Sclerosis (MS), or Parkinson’s Disease (PD) and Inpatient Asymptomatic Bacteriuria (ASB) and Urinary Tract Infection (UTI) Encounters

Variable	Overall Cohort (N = 170), No. (%)	SCI/D (N = 81, 47.6%), No. (%)	MS (N = 61, 35.9%), No. (%)	PD (N = 28, 16.5%), No. (%)
Sex, male^a^	143 (86.1)	68 (85.0)	47 (81.0)	28 (100)
**Age group^b^**				
18–49 y	15 (8.8)	13 (16.1)	2 (3.28)	0
50–64 y	47 (27.7)	23 (28.4)	23 (37.7)	1 (3.57)
65+ y	108 (63.5)	45 (55.6)	36 (59.0)	27 (96.4)
**Comorbidities^c^**				
Charlson score, median [IQR]	3 [3]	3 [3]	3 [3]	4 [4]
Cerebrovascular disease	17 (10.0)	4 (4.9)	9 (14.8)	4 (14.3)
Cardiovascular disease	20 (11.8)	9 (11.1)	8 (13.1)	3 (10.7)
Peripheral vascular disease	22 (12.9)	10 (12.4)	9 (14.8)	3 (10.7)
COPD	35 (20.6)	18 (22.2)	10 (16.4)	7 (25.0)
Chronic liver disease	9 (5.3)	5 (6.2)	3 (4.9)	1 (3.6)
Diabetes	52 (30.6)	24 (29.6)	19 (31.2)	9 (32.1)
Chronic kidney disease	46 (27.1)	18 (22.2)	17 (27.9)	11 (39.3)
Gastrointestinal ulcers	5 (2.94)	3 (3.7)	1 (1.6)	1 (3.6)
History of cancer	19 (11.2)	5 (6.2)	5 (8.2)	9 (32.1)
Dementia	29 (17.1)	4 (4.9)	10 (16.4)	15 (53.6)
Kidney stone(s)	25 (14.7)	10 (12.4)	12 (19.7)	3 (10.7)
**Bladder management**				
Catheterization	132 (77.6)	68 (84.0)	47 (77.9)	17 (60.7)
Indwelling transurethral catheter	75 (44.1)	35 (43.2)	27 (44.3)	13 (46.4)
Indwelling suprapubic catheter	29 (17.1)	13 (16.1)	14 (23.0)	2 (7.1)
Clean intermittent catheterization	28 (16.5)	20 (24.7)	6 (9.8)	2 (7.1)
Noncatheterization	38 (22.4)	13 (16.0)	14 (22.1)	11 (39.3)
External urinary device	19 (11.2)	10 (12.4)	6 (9.8)	3 (10.7)
Incontinent urinary diversion/stoma	2 (1.2)	0	1 (1.6)	1 (3.6)
Spontaneous voiding	6 (3.5)	3 (3.7)	2 (3.3)	1 (3.6)
Other (eg, incontinent into diaper)	11 (6.5)	0	5 (8.2)	6 (21.4)
**Provider type**				
Physician	153 (90.0)	70 (86.4)	57 (93.4)	26 (92.9)
Advanced practice provider	17 (10.0)	11 (13.6)	4 (6.6)	2 (7.1)
**Provider specialty**				
PM&R or SCI/D	89 (52.4)	60 (74.1)	27 (44.3)	2 (7.1)
Internal medicine	60 (35.3)	16 (19.8)	22 (36.1)	22 (78.6)
Other^d^	21 (12.4)	5 (6.2)	12 (19.7)	4 (14.3)

Note. SCI/D, spinal cord injury/disorder; MS, multiple sclerosis; PD, Parkinson’s disease; COPD, chronic obstructive pulmonary disease; PM&R, physical medicine and rehabilitation.

a Variable presented for N=166 unique patients.

b Age at encounter.

c Diagnoses recorded in the 365 days prior to the ASB or UTI encounter date.

d Overall: urology (n=3); neurology (n=7): infectious diseases (n=9); nephrology (n=1); pulmonary and critical care (n=1); SCI: ID: (n=4); pulmonary and critical care (n=1); MS: urology (n=2); neurology (n=6); infectious diseases (n=3); nephrology (n=1); PD: urology (n=1); neurology (n=1); infectious diseases (n=2).

Table 2 shows the frequency of ASB (n = 51, 30%) and UTI (n = 119, 70%) encounters with appropriate and inappropriate diagnosis and treatment categorizations. All ASB encounters had appropriate diagnoses, and 96.1% also had appropriate management (ie, no treatment). In contrast, 37 UTI encounters (31.1%) had inappropriate diagnoses. For 30 of the UTI encounters with inappropriate diagnoses (25.2%), patients had urine cultures that met the microbiologic criteria for bacteriuria but no UTI signs or symptoms and, thus, they had ASB. All of these encounters had inappropriate treatment. Of the remaining 89 UTI encounters, 31 (26.1%) had inappropriate treatment: 22 (18.5%) had inadequate antibiotic prescribing and 9 (7.6%) included overprescribing of antibiotics. Of the 81 encounters for patients with true ASB, 51 encounters had appropriate ASB diagnosis and 30 encounters had inappropriate UTI diagnosis (true ASB), and 80 (98.8%) had an associated urine culture.

**Table 2.** Encounter Types and Appropriate/Inappropriate Diagnosis and Treatment Categorizations

Diagnosis	ASB Encounters (N = 51), No. (%)	UTI Encounters (N = 119), No. (%)
Appropriate ASB diagnosis	51 (100)	…
Inappropriate ASB diagnosis	0 (0)	…
Appropriate UTI diagnosis	…	82 (68.9)
Appropriate UTI diagnosis without positive culture	…	10 (8.4)
Inappropriate UTI diagnosis	…	37 (31.1)
Inappropriate UTI diagnosis (true ASB)	…	30 (25.2)
Inappropriate UTI diagnosis without positive culture	…	7 (5.9)
**Treatment**	…	
Appropriate ASB treatment (ie, no antibiotics)	49 (96.1)	…
Inappropriate ASB treatment	2 (3.9)	…
Inappropriate UTI diagnosis (true ASB)		30 (25.2)
Appropriate UTI treatment	…	58 (48.7)
Inappropriate UTI treatment^a^	…	31 (26.1)
Inadequate antibiotic prescribing	…	22 (18.5)
Overprescribing of antibiotics	…	9 (7.6)

Note. ASB, asymptomatic bacteriuria; UTI, urinary tract infection.

a Inadequate antibiotic prescribing included no antibiotic, duration <7 d, or antibiotic(s) prescribed to which the cultured organism(s) was nonsusceptible; overprescribing of antibiotics included duration of >14 days or inappropriately broad-spectrum antibiotic in the absence of other concomitant infections or relevant allergies that may necessitate it.

Table 3 presents the frequencies of clinical outcomes, both stratified by underlying neurologic diagnosis and for all patients stratified by appropriate versus inappropriate ASB and UTI diagnosis and treatment categories. Outcomes were similar among the neurologic diagnosis groups. Patients with MS and PD had slightly higher frequencies of CDI, patients with PD had a higher frequency of AKI, and patients with SCI/D had longer postculture LOS. Patients with appropriate diagnosis also had longer postculture LOS: median days, 14 (IQR, 47) versus 7.5 days (IQR, 15) for inappropriate diagnosis.

**Table 3.** Clinical Outcomes for Patients With Neurogenic Bladder Due to Spinal Cord Injury or Disorder (SCI/D), Multiple Sclerosis (MS), or Parkinson’s Disease (PD) and Inpatient Asymptomatic Bacteriuria (ASB) and Urinary Tract Infection (UTI) Encounters

Outcome	Neurologic Diagnosis			ASB/UTI Diagnosis		ASB/UTI Treatment	
	SCI/D (n = 81, 47.6%), No. (%)	MS (n = 61, 35.9%), No. (%)	PD (n = 28, 16.4%), No. (%)	Appropriate (n = 133, 78.2%), No. (%)	Inappropriate (n = 37, 21.8%), No. (%)	Appropriate (n = 107, 62.9%), No. (%)	Inappropriate (n = 63, 37.1%), No. (%)
**90-day readmission**							
Yes	22 (27.2)	22 (36.1)	10 (35.7)	44 (33.1)	10 (27.0)	37 (34.6)	17 (27.0)
No	58 (71.6)	39 (63.9)	18 (64.3)	89 (66.9)	26 (70.3)	70 (65.4)	45 (71.4)
**CDI**							
Yes	2 (2.5)	4 (6.6)	2 (7.1)	7 (5.3)	1 (2.7)	3 (2.8)	5 (7.9)
No	79 (97.5)	57 (93.4)	27 (92.9)	126 (94.7)	36 (97.3)	104 (97.2)	58 (92.1)
**AKI**							
Yes	8 (9.9)	6 (9.8)	4 (14.3)	15 (11.3)	3 (8.1)	14 (13.1)	4 (6.4)
No	73 (90.1)	55 (90.2)	24 (85.7)	118 (88.7)	34 (91.9)	93 (86.9)	59 (93.7)
**MDRO on subsequent urine culture**							
Yes	23 (28.4)	21 (34.4)	6 (21.4)	39 (29.3)	11 (29.7)	33 (30.8)	17 (27.0)
No	58 (71.6)	40 (65.6)	22 (78.6)	94 (70.7)	26 (70.3)	74 (69.2)	46 (73.0)
Postculture LOS, median d (IQR)	16.5 (83)	10 (18)	11.5 (15)	14 (47)	7.5 (15)	14 (38)	9 (43)

Note. COPD, chronic obstructive pulmonary disease; PM&R, physical medicine and rehabilitation; CDI, *Clostrioides difficile* infection; AKI, acute kidney injury; MDRO, multidrug-resistant organism.

Bivariate analyses testing associations between appropriate and inappropriate ASB/UTI management and clinical outcomes are presented in Supplementary Table 4. These analyses were limited to 142 patients (83.5%), with SCI/D or MS because patients with PD represented the smallest proportion of the cohort and generally differed from patients with SCI/D or MS regarding important clinical and encounter characteristics. Appropriate diagnosis was associated with significantly longer postculture LOS (median, 14 vs 7 days; *P* = .02). No other outcomes were significantly associated with appropriate versus inappropriate ASB or UTI diagnosis or treatment. A sensitivity analysis including only UTI encounters did not reveal substantially different results from the overall analysis (data not shown).

## Discussion

UTIs are among the most common causes of hospitalization in patients with NB,^4,22,23^ and it is important to assess the accuracy of UTI diagnosis and treatment, particularly in inpatient and LTC settings. Furthermore, inappropriate management of ASB and UTI is one of the most common causes of antibiotic overuse^12,24–26^ and a frequent intervention target for antimicrobial stewardship programs; however, few studies have addressed this factor in patients with NB. In this multicenter study of veterans with NB, most of whom used bladder catheters, we identified important differences in ASB and UTI management. All patients with ASB encounters had appropriate diagnosis and nearly all had appropriate management. Many of the ASB encounters occurred in patients with SCI/D hospitalized for their annual VA SCI/D evaluation with a PM&R or SCI/D provider. These providers may be more familiar with differentiating ASB from UTI and with appropriate ASB management. Furthermore, patients for whom ASB encounters occurred during a hospital stay may have been less likely to be clinically ill compared to patients hospitalized with encounters having UTI diagnoses.

In contrast, almost one-third of UTI encounters had inappropriate diagnosis, and most occurred in patients with true ASB (and all of these had inappropriate antibiotic treatment). This finding is consistent with results from prior studies conducted in general outpatient populations,^10^ among hospitalized veterans with urinary catheters,^11^ and among long-term care residents,^12^ which have shown that 21%–50% of UTI diagnoses occur in patients with true ASB and are associated with inappropriate treatment. Unnecessary ordering of urine cultures in asymptomatic patients has long been a focus of antimicrobial stewardship interventions. An expert panel recently identified best practices for diagnostic stewardship regarding UTIs, including requiring documentation of signs or symptoms to obtain a urine culture.^27^ Our study provides further support for implementing diagnostic stewardship interventions, specifically targeting hospitalized patients with NB. Given that 35% of positive urine cultures in veterans with ASB being seen for their VA annual evaluations were inappropriately treated with antibiotics,^28^ there is great potential for diagnostic stewardship to reduce inappropriate antibiotic use in this population.

In this study, nearly 30% of patients with appropriately diagnosed UTI had inappropriate antibiotic treatment. We considered up to 14 days of treatment as within the guideline-recommended range, but this may be an underestimate. Our results are consistent with prior studies in catheterized LTC residents that have shown high rates of inappropriate broad-spectrum antibiotic use and longer-than-recommended courses for UTI treatment.^25,26^ Likewise, our study also confirms results from Griffith et al,^29^ who reported that 40% of patients with positive urine cultures at an outpatient MS clinic received inappropriate antibiotic treatment.

Although frequencies of CDI and AKI were low, nearly one-third of the cohort experienced readmission with 90 days following their ASB or UTI encounter, consistent with prior studies demonstrating high rates of hospitalization in patients with chronic neurologic injury.^4,22,23^ Nearly one-third of the cohort also had an MDRO on subsequent urine culture. Patients with SCI/D have higher rates of MDROs compared to general patient populations,^30–32^ which may relate to use of invasive devices, frequent antibiotic use, and high rates of healthcare exposures. When examining associations between ASB/UTI management and clinical outcomes, we focused only on patients with SCI/D and MS because these 2 groups were similar in patient and encounter characteristics and constituted most of the cohort. We identified longer postculture LOS in SCI/D and MS patients with appropriate diagnosis, a result that was not as significant when limited to UTI encounters. Many of the ASB encounters with appropriate diagnosis were performed by PM&R or SCI/D providers during SCI annual evaluations. Given the complexity and amount of care delivered during these hospitalizations, lengths of stay can be quite prolonged for reasons that may be unrelated to an ASB or UTI episode, which may account for our observed association.

Our study had several limitations. We used VA data, which may not be fully representative of patients with NB cared for outside the VA. Our results were limited by documentation in provider notes and other sources in the VA electronic health record. Identifying UTI signs and symptoms in patients with impaired bladder function and sensation can be difficult and may have impacted the accuracy of our diagnosis categorizations. We used standardized UTI signs and symptoms from national guidelines and included non–urinary-tract–specific symptoms such as fever or altered mental status when it was clear that these symptoms were not due to an alternate etiology. Our categorization of patients with UTI symptom(s) but without urine cultures meeting microbiologic criteria as ‘appropriate UTI diagnosis’ may have overestimated the number of patients with appropriate UTI diagnosis. Because this categorization was only 12% of all UTI encounters, we expect any overestimate to be minimal. Finally, due to small sample size and some infrequent outcomes, we could not perform adjusted multivariable models, and our results may have residual confounding.

In conclusion, to our knowledge, this is the first study evaluating appropriateness of ASB and UTI management among inpatients with NB. We identified a high proportion of encounters with a UTI diagnosis where management was inappropriate, highlighting the importance of prioritizing care settings such as inpatient rehabilitation, LTCF, and SCI/D units for antimicrobial stewardship initiatives related to ASB and UTI. Associations between inappropriate ASB and UTI management and clinical outcomes likely reflect a complex relationship impacted by various patient, institutional, and provider factors. Future work with a larger cohort examining these interactions may provide additional data on how improved ASB and UTI management in hospital settings may contribute to improved clinical outcomes.

## Supplementary material

For supplementary material accompanying this paper visit https://doi.org/10.1017/ash.2022.348. click here to view supplementary material
