# Supplementary material for: Clinical outcomes associated with guideline-discordant management of asymptomatic bacteriuria and urinary tract infection in hospitalized patients with neurogenic bladder
Source: Antimicrob Steward Healthc Epidemiol. 2022 Dec 9;2(1):e195. doi: 10.1017/ash.2022.348 (PMC9879921; doi:10.1017/ash.2022.348)
Supplement: Supplementary file 1 [file S2732494X22003485sup001.docx]

**Supplemental Table 1. Cohort definitions**

| **Diagnosis** | **ICD-10-CM codes** | **Definition** |
| --- | --- | --- |
| SCI/D |  | VA SCI/D Registry* |
| MS | G35 | ≥3 codes in one year  OR  ≥1 code and ≥1 MS-specific disease modifying therapy** prescribed in one year |
| PD | G20 | ≥2 codes within 3 months  AND  ≥1 PD medication*** prescribed during study period |
| NB | N31.x, N32.81 | ≥2 codes that are a minimum of 2 months apart  OR  ≥2 prescriptions for ≥ 1 bladder management supply (e.g., catheters) during the study period |
| UTI | N30.0x, N30.9x, N39.0, T83.51x | Any encounter with at least 1 code |

* Abbreviated summary of inclusion criteria: patients assigned to a SCI Patient Aligned Care Team or with utilization in an SCI/D treating specialty or with a SCI/D annual exam or with at least 2 encounters in an SCI outpatient clinic, telephone clinic, telehealth Virtual clinic, or Home Care program with a qualifying SCI/D diagnosis on 2 different calendar days. Included patients are also required to have had utilization in at least 2 of 10 workload categories derived from CDW identified patient encounters for various SCI care settings or with primary or secondary qualifying SCI and MS diagnoses (via ICD9 and 10 codes

**Glatiramer acetate, Interferon-beta 1b, Interferon-beta 1a, Natalizumab, Fingolimod, Dimethyl fumarate, Teriflunomide, Ocrelizumab, Siponimod

***Levodopa-carbidopa, Carbidopa-levodopa-entacapon, Carbidopa, Levodopa, Levodopa-Benserazide, Pramipexole, Ropinirole, Pergolide, Rotigotine, Cabergoline, Monoamine Oxidase B (MAO-B) inhibitors

**Supplemental Table 2. ASB and UTI diagnosis categorizations**

|  | **Signs or symptoms criterion** | **Microbiologic criterion** |
| --- | --- | --- |
| **UTI Diagnosis** | | |
| Appropriate | ≥1 of the following signs and/or symptoms:  Temperature >38.0°C or 100.4°F^a^  Rigors^a^  Altered mental status^a^  Malaise, lethargy, or sense of unease^a^  Flank pain  Pelvic pain or discomfort  Acute hematuria  Increased spasticity^a^  Increased autonomic dysreflexia^a^  Discomfort or pain during urination  New onset or increase in urinary incontinence | Urine culture performed within ± 7 days of the encounter grew ≥10^^3^ cfu/ml of ≥1 bacterial species |
| Appropriate without positive culture | ≥1 of the above signs and/or symptoms | Urine cultures not meeting microbiologic criterion or no urine culture within ± 7 days of the encounter |
| Inappropriate—true ASB | No signs or symptoms | Urine culture performed within ± 7 days of the encounter grew ≥10^^5^ cfu/ml of ≥1 bacterial species |
| Inappropriate without positive culture | No signs or symptoms | Urine cultures not meeting microbiologic criterion or no urine culture within ± 7 days of the encounter |
| **ASB Diagnosis** | | |
| Appropriate | No signs or symptoms | Urine culture performed within ± 7 days of the encounter grows ≥10^^5^ cfu/ml of ≥1 bacterial species |
| Inappropriate – true UTI | ≥1 of the above signs and/or symptoms | Urine culture performed within ± 7 days of the encounter grew ≥10^^3^ cfu/ml of ≥1 bacterial species |

^a^With no alternate better explanation identified for these symptoms.

**Supplemental Table 3. ASB and UTI treatment categorizations**

|  | **Criteria** |
| --- | --- |
| **UTI Treatment** | |
| Appropriate | An antibiotic prescribed ± 5 days from the culture date that meets all three criteria:   1. The cultured organism(s) is susceptible to the antibiotic selected 2. The antibiotic penetrates the urinary tract with proven efficacy for UTI 3. The antibiotic selected was prescribed for 7-14 days |
| Inappropriate | An antibiotic prescribed including ± 5 days from the culture date that does not meet all three criteria above  OR  No antibiotic prescribed |
| Inappropriate UTI diagnosis -- True ASB | Any antibiotic prescribed within ± 5 days from the culture date |
| Overprescribing | Antibiotic duration of >14 days  OR  Inappropriately broad-spectrum antibiotic in the absence of other concomitant infections or relevant antibiotic allergies that may necessitate it |
| Inadequate prescribing | No antibiotic prescribed  OR  Antibiotic prescribed for <7 days  OR  Antibiotic prescribed to which the cultured organism(s) was non-susceptible |
| **ASB Treatment** | |
| Appropriate | No antibiotic prescribed ± 5 days from the culture date  OR  An antibiotic prescribed ± 5 days from the culture date but for a suspected infection outside the urinary tract |
| Inappropriate | Any antibiotic prescribed within ± 5 days from the culture date |

**Supplemental Table 4. Bivariate analysis of associations between ASB and UTI diagnosis and treatment groups and clinical outcomes in patients with NB due to SCI/D or MS (n=142 encounters)**

|  | **ASB/UTI Diagnosis** | | | | **ASB/UTI Treatment** | | | |
| --- | --- | --- | --- | --- | --- | --- | --- | --- |
| **Outcome** | Appropriate (n=110, 77.5%) | Inappropriate (n=32, 22.5%) | OR (95% CI) | p-value | Appropriate  (n=87, 61.3%) | Inappropriate (n=55, 38.7%) | OR (95%CI) | p-value |
| **90-day readmission** |  |  |  |  |  |  |  |  |
| Yes | 34 (30.9) | 10 (32.3) | 0.94 (0.40-2.2) | 0.89 | 29 (33.3) | 15 (27.3) | 1.3 (0.62-2.7) | 0.49 |
| No | 76 (69.1) | 21 (67.7) | ref |  | 58 (66.7) | 39 (70.9) | ref |  |
| **CDI** |  |  |  |  |  |  |  |  |
| Yes | 6 (5.4) | 0 | - | - | 3 (3.4) | 3 (5.4) | 0.62 (0.12-3.2) | 0.57 |
| No | 104 (94.6) | 32 (100) | ref |  | 84 (96.6) | 52 (94.6) | ref |  |
| **AKI** |  |  |  |  |  |  |  |  |
| Yes | 12 (10.9) | 2 (6.2) | 1.8 (0.39-8.7) | 0.42 | 11 (12.6) | 3 (5.4) | 2.5 (0.67-9.4) | 0.15 |
| No | 98 (89.1) | 30 (93.8) | ref |  | 76 (87.4) | 52 (94.6) | ref |  |
| **MDRO on subsequent urine culture** |  |  |  |  |  |  |  |  |
| Yes | 33 (30.0) | 11 (34.4) | 0.82 (0.35-1.9) | 0.64 | 27 (31.0) | 17 (30.9) | 1.0 (0.48-2.1) | 0.99 |
| No | 77 (70.0) | 21 (65.6) | ref |  | 60 (69.0) | 38 (69.1) | ref |  |
| **Post-culture LOS, median days (IQR)** | 14 (57) | 7 (17) | - | 0.02 | 14 (46) | 9 (43) | - | 0.18 |

# OR, odds ratio; CI, confidence interval; CDI, *C. difficile* infection; AKI, acute kidney injury; MDRO, multidrug-resistant organism; LOS, length of stay; IQR, interquartile range
